# Perceived AI support and work outcomes: testing the mediating role of job crafting in AI-supported work

**DOI:** 10.3389/fpsyg.2026.1824712

**Published:** 2026-10-09

**Authors:** Fabian Willemsen, Charlotte Hopp, Susanne Mütze-Niewöhner, Verena Nitsch

**Affiliations:** Institute of Industrial Engineering and Ergonomics, RWTH Aachen University, Aachen, Germany

**Keywords:** AI adoption, attitudes, job crafting, job demands-resources model, perceived AI support, perceived productivity, wellbeing, work design

## Abstract

**Introduction:**

Organizations increasingly adopt Artificial Intelligence (AI) to gain competitive advantages, but anticipated benefits may not materialize when employee needs and work design aspects are neglected. Job crafting—employees' self-initiated work adjustments—may represent an important mechanism linking perceived AI support with work outcomes, but its role in the context of AI-supported work remains underexplored. Drawing on job demands-resources theory, this cross-sectional study examines relationships between perceived AI support, approach and avoidance crafting, and how these pathways may mediate associations with job satisfaction, perceived productivity, work engagement, turnover intentions, and burnout.

**Methods:**

Data were collected via an online survey of employees engaged in information and knowledge work in Germany, who use AI systems (*n* = 695). Using structural equation modeling, a mediation model with approach and avoidance crafting as parallel mediators was tested.

**Results:**

Results revealed that perceived AI support was positively associated with both approach and avoidance crafting. Approach crafting emerged as a dominant pathway, being positively associated with job satisfaction, work engagement, and perceived productivity and negatively associated with burnout and turnover intentions. Avoidance crafting showed contrary associations. Total effects of AI support through approach and avoidance crafting indicated associations with increased job satisfaction, perceived productivity and work engagement, and reduced burnout.

**Discussion:**

These findings extend job crafting theory to AI-supported work by indicating that perceived AI support functions as a job resource that is associated with distinct crafting pathways. Organizations should implement AI systems that employees perceive as supportive, thereby providing a resource that enables job crafting, and further promote approach over avoidance crafting through corresponding work design measures and targeted personnel development.

## 1 Introduction

Recent advancements in digital transformation and automation have fundamentally altered work and workplaces across various industries (Parker and Grote, 2022a). Following the widespread uptake of artificial intelligence (AI) applications such as ChatGPT, Claude, and Gemini, the subject of AI and generative AI in particular has gained

significant popularity among the general public and companies alike (Bankins et al., 2024). Amongst other effects, the ongoing integration of various forms of AI technologies has served as a catalyst for workplace automation, impacting a wide range of sectors, tasks and workers (Bankins et al., 2024; Milanez, 2023).

When implemented effectively, AI systems have the potential to promote favorable outcomes and offer competitive advantages for organizations (Chaudhuri et al., 2024; Enholm et al., 2022). To achieve this, it appears necessary that AI systems should be tailored to employee needs (Bankins et al., 2024) and be developed in participatory processes (Mütze-Niewöhner et al., 2022; Richthofen et al., 2022). However, new technology is frequently introduced as a one-size-fits-all solution, driven primarily by technological innovation rather than considering work design principles or requirements of human workers (Challenger et al., 2013; Parker et al., 2019). As a result, companies utilizing a technology-centric approach oftentimes fail to realize the full potential of AI systems (Bankins et al., 2024).

From a human factors and work design perspective, such technological implementations may also have unintended negative consequences for employees. As AI systems are increasingly being integrated into work environments, and become capable of automating complex, non-routine cognitive tasks involving analytical and rational knowledge (Richthofen et al., 2022), employees' perceptions of work and job security are changing (Bankins et al., 2024; Parker and Grote, 2022a). The implementation of AI systems has the potential to partially or fully automate existing tasks, modify current roles and responsibilities, or generate novel tasks with new skill requirements-ultimately influencing how employees perceive their work tasks and meaningfulness. For example, when AI systems automate tasks perceived as meaningful, employees may experience diminished person-job fit, job meaningfulness, reduced wellbeing, or dissatisfaction (Nazareno and Schiff, 2021). Furthermore, the perception of AI can give rise to adverse outcomes including psychological distress or concerns regarding technology substitution (e.g., Presbitero and Teng-Calleja, 2023), particularly in knowledge-intensive jobs, which in previous technological shifts had been considered relatively insulated from AI-driven changes (Chowdhury et al., 2022; Sowa et al., 2021). Notably, employees' perceptions of these changes are shaped by individual factors, and AI introduction may not only evoke resistance but, in extreme cases, also resignation in the form of turnover intentions (Chiu et al., 2021).

Consequently, the adoption of AI systems influences not only work design but also employees' perceptions and behaviors, and the extent to which investments in AI contribute to desired work-related outcomes such as worker wellbeing, innovation, and productivity (Bankins et al., 2024; Parker and Grote, 2022a; Verma and Singh, 2022). In the case of algorithmic management, the integration of algorithms and AI to perform management functions, such as scheduling or performance monitoring, can have negative impacts on workers, particularly when job demands are high and resources low (Parent-Rocheleau and Parker, 2022; Parker and Grote, 2022b), for example, when skill requirements increase while decision-making autonomy decreases. These findings underscore that the effects of AI systems are not deterministic but depend on how AI-supported work is designed, perceived, and used by employees (Parker and Grote, 2022a). Thus, ensuring an appropriate balance between job demands and job resources is essential to promote positive outcomes, including job satisfaction, engagement, health, wellbeing, and performance (Bankins et al., 2024; Demerouti, 2022; Parker and Grote, 2022b).

When adopting AI systems, companies must not only assess how job demands and resources are affected (e.g., autonomy, workload, performance monitoring; Parker and Grote, 2022a), but also how employees proactively shape their work in response to AI-supported work environments (Parker and Grote, 2022b), e.g., through job crafting. Job crafting refers to self-initiated changes and proactive behaviors that employees engage in to shape and adapt the characteristics of their work to create a better person-job fit (Tims and Bakker, 2010; Wrzesniewski and Dutton, 2001). In contrast to traditional top-down approaches to work design-in which managers define tasks and employees carry them out-job crafting emphasizes the active role of employees in shaping their own work context, complementing management-driven work design (Oldham and Hackman, 2010; Parker and Grote, 2022b; Tims and Bakker, 2010; Wrzesniewski and Dutton, 2001). Moreover, recent survey evidence shows that some employees integrate AI systems into their work independently, without formal organizational adoption (Arntz et al., 2025). As such, job crafting might play an important role in the adoption process of AI technology (Demerouti, 2022; Parker and Grote, 2022a; Parker and Knight, 2024).

Although previous research has investigated predominantly positive effects of (some forms of) job crafting on individual and organizational outcomes (Lichtenthaler and Fischbach, 2019; Rudolph et al., 2017; Zhang and Parker, 2019), less is known about how job crafting applies to work with AI technology (Parker and Grote, 2022a). With a focus on information and knowledge work, the present study investigates the role of job crafting in the context of AI-driven transformation, and how it may convey benefits. Specifically, we examine how employees' perceived AI support relates to job crafting behaviors, and how job crafting effects apply in AI-supported work contexts including associations with favorable outcomes (job satisfaction, perceived productivity, and work engagement), and adverse outcomes (turnover intentions and burnout). In doing so, we explore the importance of employee-driven design of AI-supported work and contribute to job crafting theory by testing a dual-pathway model that distinguishes between approach-oriented and avoidance-oriented crafting and their differential effects on employee outcomes.

## 2 Theoretical background

### 2.1 Job crafting as a bottom-up work design mechanism

In recent years, job crafting has emerged as an influential concept in work and organizational psychology. Wrzesniewski and Dutton (2001) originally defined job crafting in their role-based conceptualization as physical and cognitive changes employees make in their task or relational boundaries in order to align their work with their own needs, skills, and preferences. In line with this basic principle, Tims and Bakker (2010) embedded job crafting in the context of the Job Demands-Resources (JD-R) Model and view it as a mechanism through which employees can actively modify their job demands and job resources, aiming to increase their own wellbeing (Tims et al., 2012). These theoretical perspectives have been integrated in recent job crafting research by distinguishing approach and avoidance crafting (Zhang and Parker, 2019): approach crafting involves behaviors aimed at achieving positive work experiences by increasing resources or challenging demands (e.g., expanding boundaries or taking on new, more complex tasks), whereas avoidance crafting involves behaviors directed toward reducing or eliminating negative aspects of one's work by decreasing hindering job demands (e.g., reducing mentally intense work activities). This differentiation is seen as an important step in mapping the factorial structure of job crafting, as approach and avoidance crafting show different effects, and job crafting as a single factor often suffers from validity issues (Lopper et al., 2024; Zhang and Parker, 2019).

In empirical studies, approach crafting has been shown to be associated with positive work outcomes and wellbeing factors, such as increased job satisfaction or meaning of work, as well as with work engagement and performance (Lichtenthaler and Fischbach, 2019; Lopper et al., 2024; Rudolph et al., 2017; Zhang and Parker, 2019). Avoidance crafting, despite theoretical assumptions of its positive effects on such factors (Tims et al., 2013), has been found to be negatively associated with work-related outcomes, including work engagement and performance (Lichtenthaler and Fischbach, 2019; Lopper et al., 2024; Rudolph et al., 2017; Zhang and Parker, 2019). This opposite pattern of relationships has also been observed for the antecedents of approach and avoidance crafting, including job resources and autonomy, which positively predict approach crafting while negatively predicting avoidance crafting (Zhang and Parker, 2019).

Against the backdrop of an increased integration of AI at the workplace, job crafting may enable employees to adapt to technology-driven changes and actively participate in shaping their work (Demerouti, 2022; Parker and Grote, 2022a,b). In a brief overview of digitalization and bottom-up work design, Parker and Grote (2022b) note that the opportunities and challenges of new technologies are often more successfully addressed through job crafting than through top-down, organization-driven work design. Accordingly, the present study conceptualizes job crafting as a key work design process through which employees proactively shape their work in AI-supported contexts. This draws on Demerouti's (2022) view that the only way to collectively profit from digitalization and automation (including AI systems) is to turn it into a resource for employees by designing technology to support people's work and enabling people to craft its use. If AI systems are introduced into workplaces and employees retain autonomy over how and when they use them to support their work, this enables AI to represent an additional job resource the use of which is subject to proactive employee behavior.

In further investigating job crafting in the context of work with AI, we focus on employees who are primarily engaged in information and knowledge work, including administrative, managerial, and office tasks characterized by information technology use and non-routine work (adopting a broader definition of knowledge work that includes employees across educational levels; cf. Pyöriä, 2005). In information and knowledge work, handling and producing information as well as using one's knowledge are substantial to the work (North and Güldenberg, 2008; Pyöriä, 2005). Knowledge workers are particularly likely to engage in and benefit from job crafting, given their usually higher autonomy and career aspirations, yet they are increasingly subjected to excessive work demands that might require proactive work design strategies (Parker, 2014). Moreover, the integration of AI systems into information work settings creates both pressure but also opportunities for job crafting (Bhargava et al., 2021; Chowdhury et al., 2022; Demerouti, 2022; Parker and Grote, 2022a). For instance, consultants or managers might react to the adoption of AI systems to maintain their role identity by engaging in different tasks, or by refocusing on customer-centric work to maintain their role identity (Perez et al., 2022; Strich et al., 2021).

### 2.2 Job crafting and AI technology

In the last few years, a number of studies have examined how job crafting applies to digitalization (Parker and Grote, 2022b), exploring how job crafting emerges in response to anticipated technology changes (e.g., perceived automation threats, Zhang et al., 2019), how digital environments enable job crafting opportunities (e.g., taking on additional assignments; Rogiers et al., 2021), and how employees use technology to proactively alter their job demands and resources (Handke et al., 2025; Tarafdar and Saunders, 2022).

In the context of AI-supported work, existing quantitative research has predominantly focused on negative AI perceptions as triggers of employee job crafting. These include perceptions of being replaced by AI in the future (Kang et al., 2023; Wang et al., 2022) and challenge and hindrance appraisals of AI (Cheng et al., 2023; He et al., 2024; Zhang et al., 2019) as antecedents of job crafting. Perceived AI explainability has been linked to job crafting through enhanced AI-oriented benefit perception and reduced AI-oriented threat perception (Huo et al., 2025).

However, few studies examine AI as a supportive resource or investigate job crafting as a mediating mechanism linking perceptions of AI systems to work outcomes. In the service context, He et al. (2024) captured AI-induced job uncertainty as an additional job demand and found that challenge appraisal toward AI had an indirect positive influence on service performance through employee job crafting, whereas hindrance appraisal showed the opposite effect.

Overall, prior research on AI and job crafting has predominantly focused on negative perceptions of AI as a demand rather than examining AI as a supportive resource. Furthermore, while effects of job crafting are well-documented in traditional contexts, job crafting outcomes in AI-supported work remain underexplored, and job crafting has rarely been considered as a mechanism mediating between perceptions of AI systems and work outcomes. Additionally, research seldom differentiates between approach and avoidance crafting when examining outcomes in AI-supported work contexts. This study addresses these gaps by examining perceived AI support as an antecedent of job crafting, exploring how job crafting operates in AI work contexts, and testing job crafting as a mediator between perceived AI support and employee outcomes.

### 2.3 Perceived AI support as a job resource

According to the JD-R model, job resources (e.g., autonomy, supervisor support) facilitate goal achievement, stimulate growth, and help employees deal with demands and changing work conditions (Bakker and Demerouti, 2007, 2017; Demerouti and Bakker, 2023). In the context of AI adoption and use, perceived AI support represents such a job resource. In this study, perceived AI support refers to employees' perceptions that an AI system is capable to relieve them from unpleasant tasks, handle supplementary activities they would not have time for, enable focus on meaningful or more important work, support creative problem-solving, and enhance overall task performance. When AI systems are designed to automate routine or cognitively demanding subtasks, synthesize information, and support decision-making, they may enhance autonomy and facilitate proactive work behavior (Demerouti, 2022; Jia et al., 2025).

Empirical work suggests that employees who perceive AI positively and view it as a resource are more likely to use it effectively and to achieve favorable performance and wellbeing outcomes (Bankins et al., 2024; Chiu et al., 2021; Rick et al., 2024; Sowa et al., 2021). Conversely, AI systems perceived as limiting autonomy or increasing demands may generate resistance, reduce engagement, and undermine anticipated benefits (Bankins et al., 2024; Chiu et al., 2021; Mayer et al., 2020). When employees perceive AI as supportive rather than threatening, they are more likely to experience the autonomy and control that are central psychological conditions for job crafting (Tims et al., 2012; Wrzesniewski and Dutton, 2001; Zhang and Parker, 2019).

Building on these considerations, we propose that employees who perceive high AI support experience sufficient autonomy and capability to actively shape their work in line with their needs and preferences. Specifically, perceived AI support-as an additional job resource-is expected to be positively associated with approach crafting by providing the means to initiate changes to one's job, while simultaneously being negatively associated with the need for avoidance crafting. However, the relationship with avoidance crafting may be more complex, as employees might utilize an implemented AI system (e.g., chatbots) to actively delegate mentally or emotionally exhausting tasks, thereby using AI to reduce hindering demands-which would align with avoidance crafting and suggest a positive relationship. Given the diversity of AI systems and use contexts, and consistent with meta-analytic evidence showing negative associations between job resources and avoidance crafting (Lichtenthaler and Fischbach, 2019; Rudolph et al., 2017; Zhang and Parker, 2019), we hypothesize:

*Hypothesis 1a*: Perceived AI support is positively associated with approach crafting.
*Hypothesis 2a*: Perceived AI support is negatively associated with avoidance crafting.

### 2.4 Relevant outcomes and the mediating role of approach and avoidance crafting

To assess the effects of job crafting in the context of AI workplaces, this study examines constructs from three domains that have been found to be significant outcomes: job attitudes, occupational wellbeing, and work performance (Rudolph et al., 2017; Zhang and Parker, 2019). These outcomes are especially relevant in AI implementation contexts, as organizational technology investments often aim to enhance not only productivity but also employee wellbeing and attitudes (Bankins et al., 2024; Chiu et al., 2021). Specifically, we focus on job satisfaction, subjectively perceived productivity, and work engagement as desired outcomes, while turnover intentions and burnout represent adverse outcomes.

Job satisfaction and work engagement capture employees' positive affective states as responses to work, which may be enhanced when an AI system is perceived as supportive (Bankins et al., 2024; Rick et al., 2024). Subjectively perceived productivity reflects employees' perceptions of their task performance and results (Mayer et al., 2023), which is a key outcome that AI implementation explicitly aims to improve (Enholm et al., 2022). Conversely, burnout and turnover intentions represent adverse outcomes that may emerge when AI adoption increases demands, reduces meaningful work, or triggers fears of being replaced (Chiu et al., 2021; Koo et al., 2021; Presbitero and Teng-Calleja, 2023; Verma and Singh, 2022).

As opportunities of new technologies may be addressed more successfully through bottom-up work design (Demerouti, 2022; Parker and Grote, 2022b), we propose that approach and avoidance crafting could serve as mediating mechanisms linking perceived AI support to these outcomes. When AI systems support work in a targeted way, enhance autonomy and facilitate proactive work behaviors, employees gain opportunities for job crafting that may not have existed previously, allowing them to align their work with their preferences. By examining this comprehensive set of outcomes, we can assess whether approach and avoidance crafting are consistent with a dual-pathway mediating mechanism through which perceived AI support is associated with enhanced work experiences. Thus, we hypothesize:

*Hypotheses 1b–f* : Approach crafting mediates the associations between perceived AI support and work outcomes, specifically, approach crafting is positively related to (H1b) job satisfaction, (H1c) perceived productivity, and (H1d) work engagement, as well as negatively related to (H1e) turnover intentions and (H1f) burnout.
*Hypotheses 2b–f* : Avoidance crafting mediates the associations between perceived AI support and work outcomes, specifically, avoidance crafting is negatively related to (H2b) job satisfaction, (H2c) perceived productivity, and (H2d) work engagement, as well as positively related to (H2e) turnover intentions and (H2f) burnout.

Recent job crafting literature conceptualizes approach and avoidance crafting as distinct, non-redundant dimensions with incremental validity, suggesting that both should be examined simultaneously to fully capture the job crafting's effects (Lopper et al., 2024; Zhang and Parker, 2019). Given our hypothesized relations between perceived AI support and the two job crafting orientations, we expect positive total effects, comprising direct effects of perceived AI support and its indirect effects through approach and avoidance crafting, on desired outcomes, and negative total effects on adverse outcomes. This is because perceived AI support, as a job resource, should primarily enable approach crafting while reducing the need for avoidance crafting (H1a, H2a), and because approach crafting typically demonstrates stronger associations with desired, positive outcomes than avoidance crafting (Lopper et al., 2024; Rudolph et al., 2017):

*Hypotheses 3a–e*: Perceived AI support shows positive total effects (through both approach and avoidance crafting pathways) on (H3a) job satisfaction, (H3b) perceived productivity, and (H3c) work engagement, as well as negative total effects on (H3d) turnover intentions and (H3e) burnout.

## 3 Materials and methods

### 3.1 Procedure

Sample data were collected via an online survey in June 2024. For acquisition purposes, a survey panel provider was commissioned, who accessed a random sample of panel members living in Germany and who met the inclusion criteria: participants had to be at least 18 years old and no older than 65 years, be employees primarily engaged in information-intensive work (e.g., administrative or management tasks, office work) and use AI systems in their work. The participation in this study was voluntary, respondents received financial compensation for their participation, and anonymity and confidentiality were strictly preserved. Participants were informed about the latter, the reason for the study and how data would be stored. All participants consented to the use of their data for research purpose. The study met a list of standard criteria in full compliance with the European Code of Ethics in Social Science in Humanities. An ethical approval was not required as the study did not meet any conditions requiring review by the local ethics committee (Interfaculty Ethics Committee of RWTH Aachen University). Specifically, written informed consent to participate in the study was provided by the participants and the study did not involve vulnerable populations, physical risks, harmful questions, intrusive measures, deception, non-invasive bodily recordings, security-relevant risks, or exposition to heightened risks through participation.

At the beginning of the survey, participants were informed that the survey aims to examine work adjustments initiated by employees themselves in the context of artificial intelligence (AI) and automation. Respondents were presented with the definition of AI-systems as machine-based systems capable of operating either independently or with human assistance, able to adapt to different situations and processes, and capable of delivering outputs such as predictions, recommendations, decisions, or content (European Parliament and Council of the European Union, 2024; Marguerita and Morgan, 2023). They were further provided with examples of AI capabilities (e.g., collection, analysis and evaluation of data, communication with other systems; capacity to execute these tasks autonomously or to provide assistance). They were asked whether they use such system in their work and whether it was introduced by their organization or adopted independently by themselves (e.g., to make their tasks or daily work easier). Participants were instructed to think of the implemented AI system for the rest of the survey and reminded to think about the system when questions were asked about the system. To reduce fatigue effects and increase participation, shortened scales of outcome variables were used where possible.

To ensure data quality, different measures were taken. Participants who did not finish the survey, or who did not pass an attention check item (Shamon and Berning, 2019) were excluded. Of the 768 participants who finished the survey, 73 were excluded from the evaluation based on recommendations to identify speeding and straightlining behaviors, suggesting insufficient effort (Leiner, 2019; Schonlau and Toepoel, 2015). To mitigate common method bias, procedural remedies were adopted (Podsakoff et al., 2003), including securing respondents' anonymity, and measuring predictors, mediators, and outcomes on separate survey pages.

### 3.2 Sample

In total, the analyzed sample (*n* = 695) consisted of 255 females (36.7%), 437 males (62.9%), and three participants who identified as non-binary (0.4%). The average age of participants was *M* = 38.64 (*SD* = 10.44) years, ranging from 18 to 65 years. The average number of working hours per week was *M* = 37.35 (*SD* = 7.07). 4.5% of the respondents held entry level, 41.7% mid-level and 52.9% held senior level positions or higher, while approximately half of the respondents (49.8%) stated that they held leadership roles. 53.1% of respondents held a bachelor's degree or higher. Additionally, participants were asked to indicate their predominant work requirements (categorization adopted from Tegtmeier, 2021): 16.4% reported routine requirements (involving repeated application of existing knowledge), 54.5% reported task-flexible requirements (involving frequent acquisition and flexible application of knowledge), 29.1% reported creativity and problem-solving requirements (involving generation of knowledge and approaches). Participants rated the overall impact of the AI systems on their work positively on average (*M* = 4.05, *SD* = 0.79), on a scale from 1 (very negative) to 5 (very positive). For detailed information on other AI-related variables in the sample (AI system categorization, AI adoption, type of change), see Table 1.

**Table 1:** Characteristics of AI systems, adoption, and type of change in the sample.

Variable	Category	*n*	%
Categorization of AI system	Data analysis and business analytics	230	33.1
	Generative AI	189	27.2
	Conversational AI	96	13.8
	Image and document recognition	95	13.7
	Prediction and planning	85	12.2
AI adoption	Organization-led AI adoption	410	59.0
	Self-initiated AI adoption	285	41.0
Type of change^*^	Changed way of performing existing tasks	417	60.0
	Changed way of collaborating with colleagues or clients	188	27.1
	Improvement of working conditions	298	42.9
	Deterioration of working conditions	84	12.1
	Addition of tasks or task components	182	26.2
	Elimination of tasks or task components	249	35.8
	New learning and development opportunities	239	34.4

^*^Participants indicated which of the listed changes they had personally experienced as a result of the introduction of the AI system (multiple responses possible).

Respondents were queried about their industry sector and occupation using open-ended text fields. The sample is dominated by participants employed in Information and Communication Technology (ICT), manufacturing, and public administration, education, and health sectors. Occupationally, it is predominantly business, administrative and STEM-related, reflecting a concentration in knowledge-intensive, technology-oriented jobs.

### 3.3 Measures

#### 3.3.1 Job crafting

Job crafting was measured using the German version of the Job Crafting Scale (JCS; Tims et al., 2012) by Lichtenthaler and Fischbach (2016). Approach Crafting was assessed using the respective subscales with five items each for increasing structural, increasing social resources, and increasing challenging demands (e.g., “I try to learn new things at work,” “I ask colleagues for advice,” “I try to make my work more challenging by examining the underlying relationships between aspects of my job”), while avoidance crafting was measured with six items for decreasing hindering demands (e.g., “I make sure that my work is mentally less intense”). Answers were given on a five-point Likert scale from 1 (*does not apply*) to 5 (*fully applies*). Both scales demonstrate good internal consistency reliabilities as indicated by Cronbach's alpha (approach crafting α = 0.87; avoidance crafting α = 0.86).

#### 3.3.2 Perceived AI support

Perceived AI support was measured using items adapted from the Job Perception Inventory (Human-AI-Teaming subscale; Berretta et al., 2023). We selected items focusing on the perception of AI as supportive for one's work and developed one additional item (e.g., “When working with the AI system, I feel that the AI system relieves me of tasks I am less inclined to do myself”). Items were rated on a 5-point Likert scale from 1 (*strongly disagree*) to 5 (*strongly agree*). The final five-item scale demonstrated good reliability (α = 0.83).

Initially, nine items were tested (five positively worded; four negatively worded, reverse-coded; α = 0.68). Exploratory factor analysis (maximum likelihood extraction, Promax rotation with Kaiser normalization) indicated adequate sampling adequacy (KMO = 0.84) and sphericity (Bartlett's test: χ^2^(36) = 2811.93, *p* < 0.001) but revealed a two-factor structure attributable to method effects of negatively worded items (Berretta et al., 2023; DiStefano and Motl, 2006; see Supplementary Table A for the results and item wording). This issue is well-documented in the literature, as reverse-coded items can introduce systematic response biases ( DiStefano and Motl, 2006). To avoid measurement model misspecification, the four reverse-coded items were excluded based on theoretical and empirical grounds (Clark and Watson, 1995). The remaining items aligned with our definition of perceived AI support and were found to assess the unidimensional construct suitably, preserving content and construct validity. Moreover, the exclusion resolved the two-factor artifact, improved internal reliability, and ensured convergent validity in subsequent confirmatory factor analysis. In contrast to job crafting, which represents employees' behavior directed to change job characteristics, perceived AI support captures employees' evaluation of AI as a job resource and the extent to which an AI system provides instrumental support.

#### 3.3.3 Job satisfaction

Job satisfaction, referring to a positive emotional state that results from the appraisal of one's job and workplace experiences (Locke, 1976), was measured using two items from the Michigan Organizational Assessment Questionnaire (MOAQ; Cammann et al., 1983; e.g., “All in all, I am satisfied with my job”). Answers were given on a five-point Likert scale from 1 (*does not apply*) to 5 (*fully applies*). We initially included a third reverse-coded item, but its removal substantially improved scale reliability (α = 0.70 to 0.80) and measurement model fit. Since all items measured general job satisfaction (and not subfacets), the deletion of the reverse-coded item did not compromise construct validity but rather mitigated response biases and supported theoretical clarity (Locke, 1976; Clark and Watson, 1995). The final two-item scale demonstrated good reliability (α = 0.80).

#### 3.3.4 Perceived productivity

Perceived productivity is defined as to which degree participants personally experience productivity according to their task fulfillment (Mayer et al., 2023). It was measured by three items adapted from (Mayer et al. 2023; e.g., “When you look at your work how do you value your productivity?”), with an acceptable scale reliability (α = 0.75). Answers were given on a five-point Likert scale from 1 (*very low*) to 5 (*very high*).

#### 3.3.5 Work engagement

Work engagement, defined as positive, work-related, and fulfilling state of mind characterized by vigor, dedication, and absorption (Schaufeli et al., 2002), was measured with the subscale from the German version of the Copenhagen Psychosocial Questionnaire (COPSOQ; Lincke et al., 2021) consisting of three items (e.g., “I am full of energy at my work”) and demonstrated good reliability (α = 0.82). The items were answered on a five-point Likert scale from 1 (*never*) to 5 (*always*).

#### 3.3.6 Turnover intentions

Turnover intentions, referring to the conscious and deliberate willfulness to leave the organization (Tett and Meyer, 1993), were measured with two items of the COPSOQ and one item adapted from Junker et al. (2023; e.g., “In the past 12 months, how often have you thought about changing your job?”). The items were answered on a five-point Likert scale from 1 (*never*) to 5 (*always*). The scale demonstrated good reliability (α = 0.89).

#### 3.3.7 Burnout

Burnout (the degree of an employee's physical and mental fatigue or exhaustion; Burr et al., 2019) was measured with the subscale from COPSOQ containing three items (e.g., “How often do you feel emotionally exhausted?”). Answers were given on a five-point Likert scale from 1 (*never*) to 5 (*always*). Scale reliability was demonstrated to be good (α = 0.87).

#### 3.3.8 Control variables

We controlled for age, gender, and education level, as meta-analyses have shown possible correlations between these demographic variables and job crafting (Rudolph et al., 2017). The data set consisted predominantly of mid-career employees (age: *Mdn* = 37 years) with a higher level of education (53.1% had at least an undergraduate degree). In addition, almost two-thirds of the sample consisted of male participants. Since emerging evidence suggests that employees also add AI systems independently (Arntz et al., 2025; Willemsen et al., 2024), especially due to the availability of generative AI systems, we also checked whether it makes a difference if the AI system was added at the behest of the company or based on the employee's own initiative.

### 3.4 Analytical approach

Structural equation modeling was conducted in Mplus 8 (Muthén and Muthén, 2017) to model the multi-item latent variables and hypothesized mediation paths, following a two-step approach (Anderson and Gerbing, 1988). First, confirmatory factor analyses were conducted with maximum likelihood estimation to evaluate the measurement model. To reduce model complexity and the number of parameters, items measuring approach and avoidance crafting were parceled, as has been done before by similar studies assessing job crafting (Fong et al., 2021; Tims et al., 2015). The factor structure underlying the set of items in the used job crafting scale is well understood, and thus the application of parceling was considered to be appropriate (Weijters and Baumgartner, 2022). The psychometric advantage of parceling is that it results in more reliable measurement models, and particularly in larger models, the reduction in parameter estimates can improve model convergence and model stability (Little et al., 2002, 2013). Given the complexity of our model with numerous indicators, particularly for job crafting, parceling mitigates potential estimation challenges while maintaining theoretical construct structure. Moreover, the present study proposes hypotheses regarding approach and avoidance crafting consistent with prior studies that group the approach- and avoidance-oriented strategies of the JCS into this broader distinction (e.g., Fong et al., 2021; Junker et al., 2023). In line with the approach-avoidance distinction of job crafting (Zhang and Parker, 2019), the three approach-oriented subdimensions were treated as theoretically specified, complementary facets of the higher-order approach crafting factor, rather than as differential constructs with specific hypotheses. Especially when the goal is to assess relationships of the higher-order construct rather than differential relations of the individual facets, parceling of the three distinct facets is justified (Little et al., 2013), which is the case in the present study. Concretely, approach crafting was modeled with three indicators representing the means of its subscales (increasing structural job resources, increasing social job resources, and increasing challenging job demands), whereas avoidance crafting was modeled using three indicators formed based on balanced item parceling of the reducing hindering demands subscale (Weijters and Baumgartner, 2022).

Second, to test the hypothesized relationships, the structural model was estimated using maximum likelihood estimation. We tested a parallel mediation model in which approach and avoidance crafting mediate the relationship between perceived AI support and the five outcomes. The model included direct effects of perceived AI support on all outcomes, permitting tests of partial mediation. In addition to *p*-values, bias-corrected bootstrap confidence intervals (95% *CI*) were computed based on 5,000 samples to assess the significance of effects. Control variables (age and education level as continuous variables; gender dummy coded, 1 = male; AI adoption dummy coded, 1 = organizational AI adoption) were included as manifest predictors.

## 4 Results

### 4.1 Descriptive statistical analysis

Descriptive statistics, reliability, and validity values can be found in Table 2. Table 3 depicts correlations between all variables of the measurement model.

**Table 2:** Descriptive statistics and scale reliabilities.

Variable	*M*	*SD*	α
Perceived AI support	3.80	0.78	0.83
Approach crafting	3.74	0.60	0.87
Avoidance crafting	3.24	0.88	0.86
Job satisfaction	4.08	0.78	0.80
Perceived productivity	4.04	0.59	0.75
Work engagement	3.69	0.77	0.82
Turnover intentions	2.45	1.13	0.89
Burnout	2.82	0.95	0.87

*n* = 695. α, Cronbach's alpha. For parceled scales: approach crafting α = 0.71. avoidance crafting α = 0.81.

**Table 3:** Correlations among the study variables.

Variable	1	2	3	4	5	6	7	8	9	10	11
1. Age	—										
2. Gender	−0.041	—									
3. Education	−0.132^**^	0.091^*^	—								
4. AI Adoption	−0.138^**^	0.068	0.036	—							
5. Perceived AI support	−0.029	0.071	0.166^**^	0.052	—						
6. Approach crafting	−0.142^**^	0.055	0.199^**^	−0.011	0.484^**^	—					
7. Avoidance crafting	−0.285^**^	0.074	0.077^*^	0.079^*^	0.265^**^	0.356^**^	—				
8. Job satisfaction	0.090^*^	0.023	0.138^**^	−110^**^	0.308^**^	0.492^**^	0.007	—			
9. Perceived productivity	0.098^**^	−0.006	0.130^**^	−0.104^**^	0.314^**^	0.462^**^	0.049	0.519^**^	—		
10. Work engagement	−0.013	−0.007	0.143^**^	−0.064	0.396^**^	0.601^**^	0.147^**^	0.646^**^	0.516^**^	—	
11. Turnover intentions	−0.300^**^	0.032	0.056	0.150^**^	0.044	0.068	0.396^**^	−0.387^**^	−0.246^**^	−0.152^**^	—
12. Burnout	−0.099^**^	−0.064	−0.035	0.054	−0.092^*^	−0.122^**^	0.130^**^	−0.395^**^	−0.252^**^	−0.252^**^	0.483^**^

*n* = 695. ^**^p < 0.01; ^*^p < 0.05. Age and education (no degree to doctorate) as continuous variables. Gender (1, male) and AI adoption dummy coded (1, organizational AI adoption).

### 4.2 Measurement model

The proposed measurement model (eight factors: perceived AI support, approach crafting, avoidance crafting, job satisfaction, work engagement, turnover intentions, perceived productivity, and burnout symptoms) showed acceptable fit to the data (χ^2^(247) = 904.50, *p* < 0.001; CFI = 0.927; RMSEA = 0.062; SRMR = 0.061) given its complexity and large sample size (Marsh et al., 2004). The model did not include *post-hoc* modifications (e.g., error correlations) to prioritize generalizability (Hermida, 2015). All indicators loaded significantly on their intended latent variables (all *p* < 0.001; most standardized loadings > 0.60). All constructs demonstrated adequate internal consistency (composite reliability > 0.70), convergent validity (average variance extracted > 0.50), and largely adequate discriminant validity based on the Fornell-Larcker criterion (Fornell and Larcker, 1981), though some constructs showed high correlations reflecting conceptual proximity. Results of the confirmatory factor analysis can be found in Supplementary Table B. To further assess the discriminant validity, Heterotrait-Monotrait (HTMT) ratios were calculated (see Supplementary Table C). All HTMT ratios were below the conservative threshold of 0.85, delivering evidence for discriminant validity (Henseler et al., 2015). Additionally, Harman's one-factor test with all indicators was conducted using exploratory factor analysis (principal axis factoring, unrotated) to screen for common method bias (Podsakoff et al., 2003). The factor explained only 25.90% of the total variance among the measures, which is below the commonly cited 50% threshold, suggesting common method variance is less likely to influence the relationships between the measures. However, potential common method variance should still be considered while interpreting inferential associations. Further collinearity diagnostics indicated no problematic multicollinearity with VIF values ranging from 1.160 to 1.409 (conservative threshold of < 5; O'brien, 2007). Collectively, these results support the psychometric adequacy of the constructs in the measurement model, and support the use of the eight-factor structure for subsequent structural analyses.

### 4.3 Structural model

The proposed model showed acceptable fit to the data (χ^2^(320) = 1,085.127, *p* < 0.001; CFI = 0.917; RMSEA = 0.059; SRMR = 0.063), considering its complexity and large sample size (Marsh et al., 2004). While the CFI value falls just above conventional thresholds, the model's fit remains acceptable as other fit indices are within acceptable to good ranges (Marsh et al., 2004). Standardized path coefficients and indirect effect estimates are presented in Table 4. Figure 1 depicts the hypothesized dual-pathway mediation model with standardized regression coefficients. For clarity, neither the figure nor the table includes estimates of the control variables, but significant effects are described below (for detailed effects of the control variables, see Supplementary Table D). In the following, we report standardized path coefficients, which represent effect sizes, indicating that a one standard-deviation change in the predictor is associated with a β standard-deviation change in the outcome variable (Flora et al., 2025). For indirect effects, the coefficient represents the mediated relationship between predictor and outcome via the specified mediators. Due to the cross-sectional study design, all observed effects are associative in nature and do not imply causal mediation.

**Table 4:** Results of the dual-pathway mediation model.

Effects	*B*	β	*SE*	*z*	*p*	95% CI [LL, UL]
Job crafting (APC *R^2^* = 0.42, AVC *R^2^* = 0.18)						
Direct effect: PAIS → APC	0.390	0.626	0.048	8.125	< 0.001	[0.307, 0.492]
Direct effect: PAIS → AVC	0.376	0.296	0.063	5.968	< 0.001	[0.255, 0.501]
Job satisfaction (*R^2^* = 0.49)						
Direct effect: APC → JS	1.223	0.708	0.153	8.006	< 0.001	[0.943, 1.534]
Direct effect: AVC → JS	−0.137	−0.161	0.036	−3.789	< 0.001	[−0.209, −0.066]
Indirect effect: PAIS → APC → JS	0.476	0.443	0.083	5.728	< 0.001	[0.339, 0.665]
Indirect effect: PAIS → AVC → JS	−0.051	−0.048	0.017	−3.034	0.002	[−0.092, −0.024]
Total indirect effect: PAIS → JS	0.425	0.395	0.082	5.209	< 0.001	[0.286, 0.609]
Total effect: PAIS → JS	0.401	0.373	0.058	6.864	< 0.001	[0.293, 0.524]
Perceived productivity (*R^2^* = 0.50)						
Direct effect: APC → PROD	0.879	0.717	0.121	7.262	< 0.001	[0.656, 1.122]
Direct effect: AVC → PROD	−0.061	−0.101	0.026	−2.383	0.016	[−0.113, −0.012]
Indirect effect: PAIS → APC → PROD	0.342	0.449	0.069	4.975	< 0.001	[0.231, 0.501]
Indirect effect: PAIS → AVC → PROD	−0.023	−0.030	0.011	−2.129	0.028	[−0.048, −0.005]
Total indirect effect: PAIS → PROD	0.319	0.419	0.068	4.694	< 0.001	[0.207, 0.475]
Total effect: PAIS → PROD	0.300	0.394	0.045	6.654	< 0.001	[0.215, 0.390]
Work engagement (*R^2^* = 0.59)						
Direct effect: APC → WE	1.110	0.777	0.122	9.112	< 0.001	[0.890, 1.364]
Direct effect: AVC → WE	−0.038	−0.054	0.029	−1.300	0.188	[−0.096, 0.019]
Indirect effect: PAIS → APC → WE	0.433	0.487	0.067	6.429	< 0.001	[0.320, 0.586]
Indirect effect: PAIS → AVC → WE	−0.014	−0.016	0.012	−1.219	0.215	[−0.041, 0.006]
Total indirect effect: PAIS → WE	0.418	0.471	0.067	6.205	< 0.001	[0.307, 0.575]
Total effect: PAIS → WE	0.414	0.465	0.049	8.487	< 0.001	[0.323, 0.517]
Turnover intentions (*R^2^* = 0.27)						
Direct effect: APC → TI	−0.429	−0.171	0.181	−2.372	0.021	[−0.796, −0.075]
Direct effect: AVC → TI	0.510	0.416	0.061	8.320	< 0.001	[0.394, 0.631]
Indirect effect: PAIS → APC → TI	−0.167	−0.107	0.078	−2.137	0.031	[−0.343, −0.034]
Indirect effect: PAIS → AVC → TI	0.192	0.123	0.041	4.729	< 0.001	[0.121, 0.281]
Total indirect effect: PAIS → TI	0.025	0.016	0.088	0.281	0.777	[−0.155, 0.197]
Total effect: PAIS → TI	0.038	0.024	0.064	0.595	0.548	[−0.084, 0.167]
Burnout (*R^2^* = 0.10)						
Direct effect: APC → BO	−0.458	−0.243	0.145	−3.170	0.001	[−0.744, −0.182]
Direct effect: AVC → BO	0.180	0.195	0.047	3.865	< 0.001	[0.090, 0.275]
Indirect effect: PAIS → APC → BO	−0.178	−0.152	0.061	−2.907	0.003	[−0.316, −0.074]
Indirect effect: PAIS → AVC → BO	0.068	0.058	0.021	3.249	0.001	[0.033, 0.116]
Total indirect effect: PAIS → BO	−0.111	−0.095	0.065	−1.712	0.086	[−0.248, 0.004]
Total effect: PAIS → BO	−0.137	−0.117	0.054	−2.526	0.011	[−0.244, −0.033]

Model fit: χ^2^(320) = 1085.127, *p* < 0.001, CFI = 0.917, RMSEA = 0.059, SRMR = 0.063; *n* = 695. CI, 95% confidence interval (bias-corrected bootstrap, 5000 draws). LL, lower limit; UL, upper limit; PAIS, Perceived AI support; APC, Approach crafting; AVC, Avoidance crafting; JS, Job satisfaction; PROD, Perceived productivity; WE, Work engagement; TI, Turnover intentions; BO, Burnout. All direct effects from PAIS to outcomes (not shown in table) were non-significant (all *p* > 0.05). All endogenous variables were controlled for age, gender (male = 1), education level (continuous, no degree to doctorate) and AI adoption (1, organizational AI adoption).

**Figure 1:**
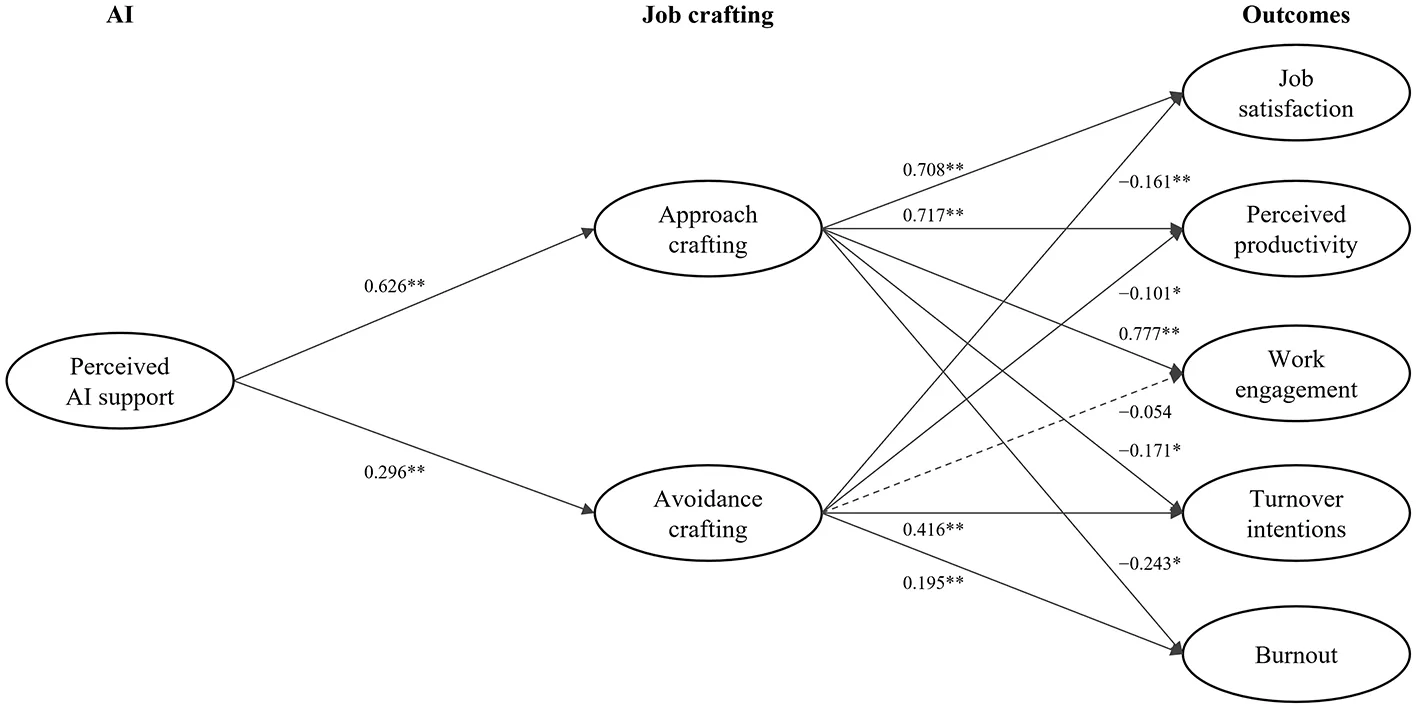
Hypothesized structural model with standardized path coefficients. **p < 0.01. *p < 0.05. Solid lines indicate significant paths; dashed line indicates a non-significant path.

Perceived AI support showed a significant positive effect on approach crafting (β = 0.626, *p* < 0.001), supporting Hypothesis 1a. Approach crafting was significantly positively related to job satisfaction (β = 0.708, *p* < 0.001), perceived productivity (β = 0.717, *p* < 0.001), and work engagement (β = 0.777, *p* < 0.001), and significantly negatively related to turnover intentions (β = −0.171, *p* = 0.021) and burnout (β = −0.243, *p* = 0.001). Consistent with Hypotheses 1b–f, significant indirect effects show that approach crafting statistically mediated the associations between perceived AI support and job satisfaction (β = 0.443, *p* < 0.001), perceived productivity (β = 0.449, *p* < 0.001), work engagement (β = 0.487, *p* < 0.001), turnover intentions (β = −0.107, *p* = 0.031), and burnout (β = −0.152, *p* = 0.003). With regard to effect sizes, direct and indirect effects (reflecting associations) on job satisfaction, perceived productivity, and work engagement were comparatively strong, while effects on turnover intentions and burnout were smaller.

Contrary to Hypothesis 2a, although perceived AI support showed a significant effect on avoidance crafting, perceived AI support was significantly positively associated rather than negatively associated with avoidance crafting (β = 0.296, *p* < 0.001). Avoidance crafting was not significantly negatively related to work engagement (β = −0.054, *p* = 0.188). However, avoidance crafting was significantly negatively related to job satisfaction (β = −0.161, *p* < 0.001) and perceived productivity (β = −0.101, *p* = 0.016), and significantly positively related to turnover intentions (β = 0.416, *p* < 0.001) and burnout (β = 0.195, *p* < 0.001). Significant indirect effects show that avoidance crafting statistically mediated the associations between perceived AI support and job satisfaction (β = −0.048, *p* = 0.002) and perceived productivity (β = −0.030, *p* = 0.028), turnover intentions (β = 0.123, *p* < 0.001), and burnout (β = 0.058, *p* = 0.001). This is in line with Hypotheses 2b, c, e, and f. No significant indirect effect was found for the association between perceived AI support and work engagement through avoidance crafting (β = – 0.016, *p* = 0.215), and thus, no support for Hypothesis 2d was found. Regarding effect sizes, direct and indirect effects (reflecting associations) were predominantly small to moderate. Avoidance crafting showed the strongest relationship with turnover intentions. Indirect effects of perceived AI support via avoidance crafting on job satisfaction, perceived productivity and burnout were comparatively weak. The relationship between AI perception and approach crafting was larger compared to its relationship with avoidance crafting.

While total indirect effects (both job crafting pathways) were significantly positive for job satisfaction (β = 0.395, *p* < 0.001), perceived productivity (β = 0.419, *p* < 0.001), and work engagement (β = 0.471, *p* < 0.001), no significant total indirect effect for turnover intentions (β = 0.016, *p* = 0.777), and burnout (β = −0.095, *p* = 0.086) were found. All direct effects of perceived AI support on outcomes were non-significant (job satisfaction: β = −0.023, *p* = 0.776; perceived productivity: β = −0.025, *p* = 0.758, work engagement: β = −0.005*, p* = 0.936; turnover intentions: β = 0.008, *p* = 0.898; burnout: β = −0.026, *p* = 0.756), indicating full mediation via the two crafting pathways. In sum, total effects of perceived AI support were significantly positive for job satisfaction (β = 0.373, *p* < 0.001), perceived productivity (β = 0.394, *p* < 0.001), and work engagement (β = 0.465, *p* < 0.001), non-significant for turnover intentions (β = 0.024, *p* = 0.548), and significantly negative for burnout (β = −0.117, *p* = 0.011; supporting Hypotheses 3a–c, e; not supporting Hypothesis 3e). In terms of magnitude, total effects on job satisfaction, perceived productivity and work engagement were relatively strong, while the total effect on burnout was smaller.

Several path coefficients differed in direction or statistical significance from the corresponding correlations reported in Table 3. In particular, approach crafting showed a small positive, non-significant correlation with turnover intentions (*r* = 0.068), whereas its path coefficient was negative and significant (β = −0.171, *p* = 0.021). Similarly, avoidance crafting showed near-zero correlations with job satisfaction (*r* = 0.007) and perceived productivity (*r* = 0.049), but the corresponding path coefficients were negative and significant (β = −0.161, *p* < 0.001 and β = −0.101, *p* = 0.016, respectively). These patterns indicate statistical suppression. Given the low VIF values (< 1.409), these coefficient changes reflect conditional associations rather than instability due to multicollinearity.

Although not hypothesized, a few control variables showed significant effects. Age was significantly positively associated with job satisfaction (β = 0.106, *p* = 0.011) and perceived productivity (β = 0.135, *p* = 0.001), and negatively associated with avoidance crafting (β = −0.290, *p* < 0.001) and turnover intentions (β = −0.184, *p* < 0.001). Male employees reported lower burnout (β = −0.094, *p* = 0.018), and higher education was associated with more approach crafting (β = 0.126, *p* = 0.002). The self-initiated (in comparison to organizational) adoption of AI was negatively related to job satisfaction (β = −0.093, *p* = 0.016), perceived productivity (β = −0.083, *p* = 0.032), and positively related to turnover intentions (β = 0.094, *p* = 0.010). Other paths showed no significance (see Supplementary Table D).

## 5 Discussion

This cross-sectional study examined the role of job crafting in AI-assisted work contexts and how job crafting serves as a pathway through which perceived AI support relates to key work outcomes. While prior research has demonstrated predominantly positive effects of approach crafting on individual and organizational outcomes (Lichtenthaler and Fischbach, 2019; Rudolph et al., 2017; Zhang and Parker, 2019), less was known about the role of job crafting in AI-driven work transformation contexts, particularly when AI systems are perceived as supportive. Focusing on employees in informational and knowledge work who utilize AI in their work, we investigated perceived AI support as a job resource and examined approach and avoidance job crafting as parallel mediating pathways linking perceived AI support to job satisfaction, perceived productivity, work engagement, turnover intentions, and burnout.

Our findings reveal a dual-pathway mechanism through which perceived AI support is associated with employee outcomes. Perceived AI support was rather strongly associated with approach crafting, suggesting that employees who view AI as supportive actively expand resources and seek challenges. Approach crafting consistently related to higher job satisfaction, work engagement, and perceived productivity, and to lower burnout and turnover intentions, mediating the relationships of perceived AI support with these outcomes. These findings extend prior job crafting research by demonstrating that AI systems can function as contextual job resources that stimulate proactive work redesign.

Contrary to expectations, perceived AI support was also positively related to avoidance crafting. Rather than indicating withdrawal behavior (Hu et al., 2020), one potential interpretation is that supportive AI systems may enable employees to strategically reduce hindering demands, for instance by offloading cognitively or emotionally taxing tasks. However, in line with earlier studies avoidance crafting was associated with less favorable outcomes (e.g., Zhang and Parker, 2019), including higher burnout and turnover intentions as well as lower job satisfaction and productivity (with no significant association with work engagement). This pattern indicates that while AI support is associated with multiple forms of job crafting, these relationships differ markedly in their implications for employee wellbeing.

Importantly, total effects indicated that perceived AI support was likely to be overall beneficial, showing significant positive relationships with desirable outcomes and lower burnout. Notably, the approach crafting pathway showed substantially stronger associations than the avoidance crafting pathway with these outcomes, with these opposing pathways partially offsetting each other. This is consistent with findings of previous studies which showed that approach crafting was more strongly linked to job satisfaction, productivity, and work engagement than avoidance crafting (e.g., Rudolph et al., 2017). The absence of significant direct effects provides further evidence for the central role of job crafting-particularly approach crafting-as the mechanism through which AI support becomes impactful. Thus, it seems likely that AI systems do not improve work outcomes *per se*; rather, their impact depends on how employees perceive and actively integrate them into their work, among other factors.

These findings suggest that supportive AI systems and employees' arising job crafting opportunities may be important for realizing the full potential of AI. Employees may benefit from AI systems to a greater extent when they can use the advantages of AI systems and their gained resources for meaningful work redesign. AI adoption and automation risk making work less meaningful, less challenging and less satisfying (e.g., Nazareno and Schiff, 2021). However, when employees perceive AI positively and engage in approach crafting, they can potentially transform increased resources into enhanced wellbeing and performance. The following subsections critically discuss the results in depth, explore possible alternative explanations, and outline different theoretical implications.

### 5.1 Approach and avoidance crafting in AI contexts

In the current study, perceived AI support was associated with both approach and avoidance crafting. This contrasts with the assumption that this job resource should primarily enable approach crafting while reducing the need for avoidance crafting. Research regularly demonstrates a negative relationship between job resources and avoidance crafting (Rudolph et al., 2017; Zhang and Parker, 2019). This pattern could reflect the underlying motivational mechanisms: while job resources activate approach-oriented behaviors through motivational pathways, job demands tend to trigger strain pathways that lead to avoidance behaviors (Rudolph et al., 2017). Specifically, higher job autonomy, as a key work characteristic, has been linked to reduced avoidance demands crafting, as autonomy enhances motivation and goal-directed behavior, thereby preventing withdrawal responses (Rudolph et al., 2017; Zhang and Parker, 2019).

However, AI contexts may uniquely facilitate both crafting forms simultaneously. The adoption of AI differs significantly from traditional digitalization and automation. If employees can use AI systems to support their work, this increases their autonomy and thus their flexibility to craft their jobs, instead of just reacting to a changed environment. In AI contexts, the active task delegation to AI systems may reflect approach-oriented job crafting rather than avoidance crafting behavior. This aligns with Demerouti and Peeters (2018) finding that hindering demands can be crafted in approach-oriented ways, thereby having a beneficial effect on work engagement. The avoidance crafting factor may actually capture different types of behaviors driven by distinct motivations (Hu et al., 2020): proactive optimization of hindering demands to preserve resources and maintain person-job fit (approach-oriented), and withdrawal behavior characterized by demand reduction to avoid aversive work characteristics.

Thus, in the present study, one explanation is that perceived AI support as a resource is associated with proactive job crafting in both forms. The moderate positive correlation between approach and avoidance crafting (*r* = 0.36) suggests a shared proactive foundation, yet distinct operational mechanisms, supporting the notion that job crafting comprises interrelated proactive behaviors employees enact to enhance person-job fit (Rudolph et al., 2017; Tims and Bakker, 2010; Wrzesniewski and Dutton, 2001). However, whether avoidance crafting constitutes proactive changes in work characteristics rather than withdrawal behavior, and whether and under which conditions it yields beneficial rather than adverse effects, is still debated (Lopper et al., 2024; Parker et al., 2026; Zhang and Parker, 2019). Furthermore, given that AI use in the workplace remains a relatively recent phenomenon, the long-term consequences of AI-related job crafting remain largely unexplored. Avoidance crafting may initially coincide with unfavorable outcomes (e.g., turnover intentions), yet contribute to more sustainable work arrangements over time.

Recent research reveals that both approach and avoidance crafting can have nuanced effects, as avoidance crafting may sometimes protect employee wellbeing in the short term, while approach crafting can come at a cost over time (e.g., through increased job complexity and workload; Tims et al., 2022). Importantly, employees may need to use both strategies simultaneously, using approach crafting to gain new resources while using avoidance crafting to reduce demands to achieve positive work outcomes (Tims et al., 2022). In relation to outcomes, a study by Seppälä et al. (2020) found that the negative effects of avoidance crafting are mitigated when combined with approach crafting.

Additionally, first studies recently investigated the idea that approach and avoidance crafting are used in combination by conducting job crafting profile analyses and following a person-centered approach (Mäkikangas, 2018; Mäkikangas and Schaufeli, 2021; Zhang et al., 2025). Such profile analyses allow to investigate the nuanced impact of antecedents such as autonomy on the simultaneous use of approach and avoidance crafting (Zhang et al., 2025). Zhang et al. (2025) found that higher job autonomy was associated with the probability of individuals being classified as proactive crafters (high use of approach crafting, moderate use of avoidance crafting) or active crafters (average level of both strategies) compared to reactive crafters (low use of approach crafting, high use of avoidance crafting). These results indicate, that job autonomy may not only allow employees to engage in approach crafting, but also allow employees to combine both approach and avoidance crafting in a balanced manner to achieve desirable work outcomes (Zhang et al., 2025). Thus, adopted AI systems that are perceived as an additional resource (e.g., supportive and enabling more autonomy) may trigger both approach and avoidance crafting behaviors simultaneously, making it essential to consider the total effect.

This may be an alternative explanation for the positive correlation between perceived AI support and avoidance crafting in the current study, and challenges the assumption that avoidance crafting is inherently problematic. When AI support enables employees to engage in both proactive expansion and strategic demand reduction, the overall pattern contributes to enhanced work experiences. For instance, in the current study employees reported in text fields that they delegated work tasks to AI (e.g., text generation) while actively changing the focus of their work on creative or challenging tasks and using freed time for collaboration in their team. Thus, avoidance crafting could become less harmful when combined with approach crafting, or in other words, avoidance crafting is most effective in changing work design when it is accompanied by approach crafting (Parker et al., 2026; Zhang et al., 2025; Zhang and Parker, 2019).

A further explanation is that employees may perceive AI concurrently as both support and threat. In a study on AI adoption, (Cheng et al. 2023) found positive correlations between organizational involvement in the adoption of AI technologies and both approach and avoidance crafting. However, this relationship was mediated by challenge appraisals (predicting approach crafting) and hindrance appraisals (predicting avoidance crafting). This suggests that perceiving AI as a hindrance or threat may lead to avoidance crafting (Cheng et al., 2023; Wang et al., 2022), even when AI is generally assessed as supportive. Moreover, AI may introduce role ambiguity or skill development pressures alongside its benefits (Bankins et al., 2024), potentially triggering avoidance behaviors even among employees with positive AI perceptions. The positive correlation between perceived AI support and avoidance crafting in the current study may thus reflect that AI systems, while predominantly perceived as supportive, can simultaneously introduce new demands or uncertainties (as indicated by 12.1% of the respondents), leading to some self-protective behaviors.

However, in the current study we found overwhelming approval of the respective AI systems, as the majority of all participants evaluated the impact as positive. It may be necessary to consider a broader spectrum of relevant everyday demands that have been associated with negative outcomes and avoidance crafting (Bakker and Demerouti, 2017; Parker and Grote, 2022a; Zhang and Parker, 2019). Furthermore, individual differences may also have had a potential impact on both the prediction of approach and avoidance crafting (Lichtenthaler and Fischbach, 2019; Rudolph et al., 2017), as well as on the surveyed outcomes (Bakker and Demerouti, 2017; Tisu et al., 2020). Avoidance crafting showed substantially lower explained variance by perceived AI support (*R*^2^ = 0.18) compared to approach crafting (*R*^2^ = 0.42), implying that avoidance behaviors are driven by broader work demands (such as role ambiguity, organizational constraints, or conflicts) or individual differences. This aligns with the modest explained variance for turnover intentions (*R*^2^ = 0.27) and burnout (*R*^2^ = 0.10), and the non-significant total indirect effects of AI support on these adverse outcomes. AI adoption does not necessarily reduce pre-existing hindering demands, as even when AI relieves employees from certain tasks, underlying demands may persist. Employees who already faced high demands before implementation may continue engaging in avoidance behavior alongside approach crafting, with these persistent demands more strongly influencing these adverse outcomes.

### 5.2 Employee-initiated AI adoption

It is noteworthy that more than a third (41%) of the employees in the sample reported to have independently integrated AI systems into their work. This may reflect the growing accessibility of AI tools (e.g., ChatGPT, DeepL) and provides new considerations when investigating how job crafting applies to (AI) technology. The adoption of AI technology itself can be interpreted as employees' approach-oriented job crafting behavior to increase their job resources and to manage their demands (Bruning and Campion, 2018). However, AI adoption can extend beyond this initial crafting: Once integrated, AI systems can create new job crafting opportunities for employees (e.g., through higher resources, such as autonomy and flexibility), not only for approach crafting but also for avoidance crafting (specifically, optimizing demands crafting). This underscores the importance of simultaneously assessing approach and avoidance crafting in the context of AI-assisted work.

First qualitative studies support this perspective, indicating that employees use generative AI to proactively craft their work tasks and relationships (Mayer et al., 2025; Willemsen et al., 2024). For example, the adoption and interaction with AI systems allows employees to engage in challenging tasks and enables new skill development opportunities. With regard to information and communication technology (ICT), Handke et al. (2025) recently demonstrated that employees actively use ICT to shape their work for themselves and that this behavior is positively correlated with skill utilization and person-environment-fit. However, their study was conducted before the widespread availability of generative AI and the adoption of AI was not as wide-spread as other technologies that offer less flexibility for work redesign (Handke et al., 2025).

In our study we controlled for self-initiated (in comparison to organizational) AI adoption and found it was significantly negatively related to job satisfaction and perceived productivity, and positively related to turnover intentions. We would expect that self-initiated AI adoption should represent an approach resource crafting behavior (e.g., Bruning and Campion, 2018), which in turn should relate to positive outcomes. Here, a reverse causality explanation is plausible: Employees experiencing dissatisfaction or performance challenges may proactively adopt AI tools to address these issues. While the self-initiated AI adoption may promote beneficial long-term effects, our cross-sectional design captures only the initial state of dissatisfaction that prompted AI adoption, not the potential future improvements.

These findings regarding self-initiated AI adoption point to complex theoretical and practical implications for AI and work design. First, when employees adopt AI systems on their own, they are not dependent on top-down technology adoption and technology design (incl. subsequent technology acceptance, demands and threat perceptions), but rather integrate systems they perceive as supportive on their own. Second, employee-initiated AI adoption underlines the importance of job crafting for organizational change and technology adoption, innovation and productivity gains, as well as enhanced human-centered work characteristics. Thus, the current digital transformation including the increasing adoption of AI systems involves a dynamic and important interplay between top-down and bottom-up work design.

### 5.3 Theoretical and practical implications

Our findings provide several contributions. This study is among the first to examine job crafting in AI contexts using a dual-pathway mediation model, indicating that perceived AI support differentially relates to approach and avoidance crafting. In doing so, we address calls for research on the role of job crafting within the context of technology (Demerouti, 2022; Parker and Grote, 2022a). While approach crafting was associated as a dominant pathway linking perceived AI support to positive outcomes, avoidance crafting showed distinct and partly adverse associations. This extends job crafting theory to the domain of AI-supported work by showing that AI systems can become essential elements of employees' bottom-up work design processes. Rather than being passive recipients of technological change, employees may actively incorporate AI into their work to reshape job resources and demands. This suggests that job crafting may serves as an important bottom-up work design process linking AI implementation to individual outcomes.

Additionally, our findings contribute to JD-R theory by conceptualizing AI not merely as a technology context factor, but in form of perceived AI support as a potential job resource promoting job crafting. This suggests that AI can function similarly to traditional resources (e.g., autonomy, supervisor support) while introducing new opportunities for proactive work design. Moreover, the differing relations of approach and avoidance crafting with outcomes underscore that not all forms of job crafting are equally beneficial in AI contexts, highlighting the importance to distinguish these pathways.

In the present cross-sectional study, the results of the mediation analysis indicate that job crafting may functions as an important mechanism through which the adoption of AI systems engenders favorable outcomes. While technological capabilities are relevant, employee perceptions of AI as supportive seem to be critical. This emphasizes the importance of both organizational work design (top-down) and employee job crafting (bottom-up) in AI implementation (Parker and Grote, 2022b).

Organizations are required to create supportive work environments and empower employees to unlock the full potential of AI adoption (Bankins et al., 2024). Accordingly, organizations may achieve desired outcomes more sustainably by focusing on augmentative rather than pure automation (Bankins et al., 2024), and considering human-centered approaches (Bednar and Welch, 2020; Parker and Grote, 2022b). Specifically, perceived AI support appears to be essential for realizing benefits from both organizational and employee perspectives, by fostering opportunities for employees to align their work with individual needs and strengths. Importantly, employees' job crafting can serve as a complementary bottom-up process to managerial work design that is associated with positive outcomes in the context of AI adoption and organizational change.

However, job crafting should not be interpreted as a substitute for organizational responsibility to design human-centered work. When organizations redesign work through the adoption of AI systems, they must focus on enhancing job resources and offering manageable demands (Demerouti, 2022; Parker and Grote, 2022a), while recognizing job crafting as valuable, sometimes necessary, addition within work contexts. Conversely, automation-focused AI adoption that neglects important work characteristics or job resources such as autonomy, skill variety, role clarity, and support (e.g., Parker and Knight, 2024), risks unintended adverse outcomes (e.g., when AI systems restrict employees' autonomy, take on meaningful tasks, or increase demands).

To enable effective job crafting, employees need support to align it with their needs and goals. Companies fostering environments where employees feel supported in job crafting, including the exploration of new AI tools, benefit from positive outcomes and successful technological change. In this context, it is worth noting that while the self-initiated adoption of AI tools may come with positive effects, the unauthorized use of AI within organizations, increasingly referred to as Shadow AI, may pose governance challenges and security risks (Silic et al., 2025), underscoring the need to reduce barriers to legitimate AI adoption while ensuring cybersecurity compliance. Taken together, this may require attention to additional factors, including employees' work design competencies, managerial and supervisor support, and data security compliance, as well as training to address these factors. With regard to job crafting, organizations could incentivize employees through job crafting interventions which increase employees' awareness regarding opportunities to redesign their work (Demerouti et al., 2019). Based on our findings and as suggested by (Zhang et al. 2025), employees could be trained to engage primarily in approach crafting while utilizing moderate avoidance crafting alongside (e.g., to avoid becoming overwhelmed) to achieve positive outcomes.

### 5.4 Limitations and future directions

Several limitations should be acknowledged when interpreting these findings. First, this study relies on a cross-sectional, self-report questionnaire, raising concerns regarding common method bias, which can inflate relationships between variables due to shared method variance (Podsakoff et al., 2003). In the current study, procedural remedies were implemented in the study design to mitigate biases and Harman's one-factor test was conducted to screen for common method bias. However, it should be noted that this test has limitations (e.g., insensitivity), and method effects may still influence the results (Podsakoff et al., 2003). Accordingly, some of the observed associations between constructs may partly reflect shared method variance as a consequence of the use of self-report data collected from a single source at a single point in time. Therefore, the magnitude of the observed relationships may be inflated and should be interpreted with caution. Nevertheless, correlations and shared variance between theoretically related constructs are to be expected when they have a common cause and do not, in themselves, indicate that common method bias is a problematic explanation for the observed relationships (Conway and Lance, 2010). Self-reports are appropriate for capturing employees' perceptions, providing insights that are inherently subjective and not directly observable by external raters. In this regard, self-reports are the most relevant method for the assessment of numerous psychological concepts, including factors such as job satisfaction or perceived job characteristics (Conway and Lance, 2010). Future studies could employ, where applicable, additional measures to address common method bias, such as adding an independent marker variable (Podsakoff et al., 2003), using peer-reports from supervisors or colleagues, or incorporating objective performance indicators (Conway and Lance, 2010). Furthermore, the cross-sectional survey design precludes causal conclusions. Although the hypothesized directionality (i.e., from perceived AI support through job crafting to subsequent work outcomes) is theoretically grounded in prior job crafting research, reciprocal or reverse relationships cannot be ruled out (Petrou et al., 2012; Tims et al., 2012). For example, employees who engage in approach crafting may perceive AI systems as more supportive. Thus, longitudinal research is needed to test the mediation processes, to confirm the observed relations between variables, as well as their causal directions. This applies particularly to self-initiated AI adoption, given the need to test whether it functions as a proactive work redesign strategy with delayed benefits, and the long-term consequences of avoidance crafting in AI-assisted contexts.

Additionally, sample-related limitations should be considered. The sample consists of employees in Germany who are primarily engaged in information and knowledge work. While this focus is theoretically meaningful given that AI adoption increasingly affects knowledge-intensive tasks, it limits generalizability to other occupational groups, national contexts, or institutional environments. Although effects may transfer to AI-supported service industries (He et al., 2024) and production contexts are increasingly involving administrative and knowledge-based tasks (Richthofen et al., 2022), future research should examine whether the observed relationships generalize to other forms of work, including contexts with lower levels of autonomy or different work design characteristics. In addition, future research should address contexts with cultural differences (e.g., individualism and collectivism; Barnes et al., 2024) and variations in institutional frameworks and barriers (e.g., regulations and infrastructure) to contribute to the generalizability of our findings. Although eligible participants were randomly selected from the panel provider's pool, the study relied on a non-probability online panel sample. In sum, this limits external validity and representativeness. Moreover, a substantial proportion of participants reported having voluntarily adopted AI systems rather than using organizationally implemented tools. Employees who self-select into AI use may differ systematically in proactivity, openness to technology, or job crafting tendencies. Although the manner of AI adoption was statistically controlled for, unobserved differences cannot be fully ruled out. Longitudinal or (quasi-)experimental designs could better disentangle proactive technology adoption from subsequent job crafting processes.

It is noteworthy that certain relationships only emerged after accounting for shared variance among predictors (e.g., the zero-order correlation between avoidance crafting and job satisfaction was close to zero). While these suppression effects were statistically robust in the present sample (not attributable to multicollinearity as indicated by VIF values), they warrant cautious interpretation and replication. To identify the genuine unique effects of the exogenous variables, future studies should replicate these relationships using longitudinal designs. However, consistent with the approach-avoidance conceptualization of job crafting, the divergent effects of both orientations emerged when modeled jointly. This emphasizes the importance of conceptualizing approach crafting and avoidance crafting as distinct, yet complementary dimensions that should be assessed simultaneously in the future (Lopper et al., 2024; Zhang and Parker, 2019).

Lastly, this study did not explicitly address negative aspects of AI implementation, such as fears of being replaced by algorithms. These scenarios lie outside the scope of the present research, as we focus on the perception of AI systems as a job resource. Improperly designed AI systems that are perceived as unsupportive or disruptive may elicit avoidance reactions or require approach crafting to regain resources. Moreover, while job crafting could represent an important process linking perceived AI support to positive work experiences, adverse outcomes such as burnout and turnover intentions are also shaped by additional mechanisms not captured in the present model, such as stress related to technology or job insecurity, as well as social support and leadership behaviors (Bankins et al., 2024; Demerouti and Bakker, 2023; Parker and Knight, 2024). Future research could examine the circumstances that determine when perceived AI support leads to more approach or more avoidance crafting, and how AI interacts with broader work demands to shape job crafting patterns.

Despite these limitations, the study provides evidence that perceived AI support is linked to employee work outcomes through job crafting, highlighting the importance of bottom-up work design processes in AI-supported work environments and suggesting the need for future research.

## 6 Conclusion

This cross-sectional study examined the potential mediating role of job crafting in AI-supported work, and tested a dual-pathway model distinguishing approach and avoidance crafting and their differential associations with work outcomes. Our findings reveal that perceived AI support is associated with both approach and avoidance crafting, with approach crafting as a dominant pathway potentially linking perceived AI support to increased job satisfaction, perceived productivity and work engagement, and reduced burnout. These findings underscore that organizations should implement AI systems as a job resource, consider work design and personnel development, and create conditions that enable job crafting, with a particular emphasis on promoting approach over avoidance crafting. Conclusively, the findings strengthen the notion that the success of AI-driven work transformation depends not only on technological implementation and function, but also on whether employees perceive AI systems as supportive, and whether they can proactively reshape their work in such ways that it aligns with their abilities, needs, and interests.

## Data Availability

The raw data supporting the conclusions of this article will be made available by the authors, without undue reservation.
