# Supplementary material for: Perceived AI support and work outcomes: testing the mediating role of job crafting in AI-supported work
Source: Front Psychol. 2026 Oct 9;17:1824712. doi: 10.3389/fpsyg.2026.1824712 (PMC13690681; doi:10.3389/fpsyg.2026.1824712)
Supplement: Supplementary file 1 [file Data_Sheet_1.pdf]

Supplementary Material

Supplementary Table A: EFA results for perceived AI support, including item text and source.

Item	Item text	Item source	Factor 1	Factor 2
PAIS1	When working with the AI system, I feel that the AI system relieves me of tasks that I am less inclined to do myself.	Berretta et al. (2023)		.737
PAIS2	The AI system takes on important complementary tasks that I would otherwise not get to due to lack of time.	Berretta et al. (2023)		.684
PAIS3	Working with the AI system means that I can concentrate fully on the important aspects of my work.	Berretta et al. (2023)		.781
PAIS4	Working with the AI system allows me to develop more creative solutions that I would not have come up with on my own.	Berretta et al. (2023)		.574
PAIS5	Working with the AI system helps me complete my tasks more effectively.	Self-developed		.764
PAIS6 (inverted)	The AI system takes over tasks that I actually enjoy and would rather do myself.	Berretta et al. (2023)	.718	
PAIS7 (inverted)	Working with the AI system leads to me only doing leftover work that actually bores me.	Berretta et al. (2023)	.864	
PAIS8 (inverted)	When working with the AI, at the end of the working day I ask myself whether I actually want to continue doing this work.	Berretta et al. (2023)	.892	
PAIS9 (inverted)	Working with the AI system, I feel like it hinders me completing my tasks.	Self-developed	.731	

Note. n = 695. PAIS = Perceived AI support. Maximum likelihood extraction with Promax rotation. Loadings < .30 are suppressed for clarity. Items reproduced from Berretta et al. (2023), Table 5, under the terms of Creative Commons Attribution License, CC-BY-4.0 (https://creativecommons.org/licenses/by/4.0/). Reference: Berretta, S., Tausch, A., Peifer, C., and Kluge, A. (2023). The Job Perception Inventory: Considering human factors and needs in the design of human-AI work. Front. Psychol. 14:1128945. doi: 10.3389/fpsyg.2023.1128945

Supplementary Table B: CFA results for the eight-factor measurement model.

Variable/Indicator	Loadings	Communalities	CR	AVE
**Perceived AI support**			.835	.505
PAIS1	.721	.520		
PAIS2	.700	.490		
PAIS3	.772	.595		
PAIS4	.593	.352		
PAIS5	.754	.569		
**Approach Crafting**			.747	.500
APC1	.697	.486		
APC2	.583	.340		
APC3	.822	.675		
**Avoidance Crafting**			.864	.680
AVC1	.884	.782		
AVC2	.831	.690		
AVC3	.754	.569		
**Job satisfaction**			.805	.674
JS1	.823	.678		
JS2	.819	.671		
**Perceived productivity**			.757	.510
PROD1	.683	.466		
PROD2	.742	.550		
PROD3	.717	.514		
**Work engagement**			.819	.603
WE1	.733	.537		
WE2	.844	.712		
WE3	.748	.560		
**Turnover intention**			.889	.729
TI1	.853	.728		
TI2	.889	.791		
TI3	.814	.663		
**Burnout**			.866	.685
BO1	.762	.580		
BO2	.882	.778		
BO3	.834	.696		

Note. $n = 695$. CR = composite reliability. AVE = average variance extracted. All loadings were significant, $p < .001$.
Fit: $\chi 2(247) = 904.50$, $p < .001$; CFI = 0.927; RMSEA = 0.062; SRMR = 0.061.

Supplementary Table C: HTMT ratios for the study variables.

Variable	1	2	3	4	5	6	7	8
1. Perceived AI support								
2. Approach crafting	.648							
3. Avoidance crafting	.311	.414						
4. Job satisfaction	.370	.669	.009					
5. Perceived productivity	.419	.669	.063	.667				
6. Work engagement	.444	.790	.176	.798	.660			
7. Turnover intentions	.081	.042	.452	.459	.301	.180		
8. Burnout symptoms	.086	.175	.151	.474	.313	.304	.551	

Supplementary Table D: Direct effects of perceived AI support and control variables in the mediation model.

Effects	*B*	β	*SE*	*z*	*p*	95% *CI* [LL, UL]
**Approach Crafting**						
Age → APC	−0.003	−.066	0.002	−1.565	.118	[−0.006, 0.001]
Gender → APC	−0.009	−.010	0.035	−0.245	.807	[−0.078, 0.060]
Education → APC	0.022	.126	0.007	3.017	.003	[0.008, 0.038]
AI adoption → APC	−0.037	−.043	0.033	−1.108	.268	[−0.103, 0.027]
**Avoidance Crafting**						
Age → AVC	−0.024	−.290	0.003	−7.351	< .001	[−0.031, −0.018]
Gender → AVC	0.043	.024	0.071	0.607	.544	[−0.096, 0.185]
Education → AVC	0.000	.001	0.014	0.021	.983	[−0.027, 0.027]
AI adoption → AVC	0.046	.026	0.067	0.693	.488	[−0.082, 0.180]
**Job satisfaction**						
Perceived AI support → JS	−0.024	−.023	0.086	−0.285	.776	[−.195, 0.136]
Age → JS	0.007	.106	0.003	2.564	.010	[0.002, 0.013]
Gender → JS	0.041	.027	0.059	0.705	.481	[−0.074, 0.157]
Education → JS	0.007	.024	0.013	0.571	.568	[−0.018, 0.032]
AI adoption → JS	−0.138	−.093	0.057	−2.412	.016	[−0.255, −0.030]
**Perceived productivity**						
Perceived AI support → PROD	−0.019	−.025	0.063	−0.308	.758	[−.151, 0.096]
Age → PROD	0.007	.135	0.002	3.200	.001	[0.003, 0.011]
Gender → PROD	−0.007	−.007	0.041	−0.177	.859	[−0.084, 0.073]
Education → PROD	0.005	.021	0.010	0.478	.633	[−0.015, 0.023]
AI adoption → PROD	−0.088	−.083	0.042	−2.095	.036	[−0.174, −0.011]
**Work engagement**						
Perceived AI support→ WE	−0.005	−.005	0.061	−0.080	.936	[−.133, 0.103]
Age → WE	0.002	.026	0.002	0.645	.519	[−0.003, 0.006]
Gender → WE	−0.048	−.038	0.046	−1.04	.298	[−0.137, 0.046]
Education → WE	−0.001	−.003	0.010	−0.08	.936	[−0.021, 0.019]
AI adoption → WE	−0.064	−.052	0.045	−1.44	.150	[−0.156, 0.023]
**Turnover intentions**						
Perceived AI support → TI	0.013	.008	0.102	0.129	.898	[−.189, 0.223]
Age → TI	−0.019	−.184	0.004	−4.799	< .001	[−0.027, −0.011]
Gender → TI	−0.05	−.023	0.079	−0.628	.530	[−0.208, 0.105]
Education → TI	0.016	.036	0.017	0.977	.329	[−0.016, 0.049]
AI adoption → TI	0.204	.094	0.080	2.561	.010	[0.053, 0.364]
**Burnout**						
Perceived AI support → BO	−0.026	−.022	0.083	−0.311	.756	[−.187, 0.139]
Age → BO	−0.005	−.071	0.003	−1.717	.086	[−0.011, 0.001]
Gender → BO	−0.156	−.094	0.066	−2.364	.018	[−0.284, −0.027]
Education → BO	−0.001	−.002	0.014	−0.037	.971	[−0.028, 0.026]
AI adoption → BO	0.052	.032	0.065	0.804	.421	[−0.076, 0.178]
Model fit: $\chi^2(320) = 1085.127$, $p < .001$, CFI = .917, RMSEA = .059, SRMR = .063						

Note. $n = 695$. CI = 95% confidence interval (bias-corrected bootstrap, 5000 draws); LL = lower limit; UL = upper limit; PAIS = Perceived AI support; APC = Approach crafting; AVC = Avoidance crafting; JS = Job satisfaction; PROD = Perceived productivity; WE = Work engagement; TI = Turnover intentions; BO = Burnout. Age and education (no degree to doctorate) as continuous variables. Gender (1 = male) and AI adoption dummy coded (1 = organizational AI adoption). For other effects, see Table 4.
